# Exosomes of Antler Mesenchymal Stem Cells Improve Postoperative Cognitive Dysfunction in Cardiopulmonary Bypass Rats through Inhibiting the TLR2/TLR4 Signaling Pathway

**DOI:** 10.1155/2020/2134565

**Published:** 2020-03-26

**Authors:** Chun Yang, Shengnan Sun, Qi Zhang, Jia Guo, Tengfei Wu, Ying Liu, Min Yang, Yan Zhang, Yinghua Peng

**Affiliations:** ^1^Institute of Special Wild Economic Animal and Plants, Chinese Academy of Agricultural Sciences, Changchun, China; ^2^State Key Laboratory for Molecular Biology of Special Economic Animal, Changchun, China; ^3^Department of Laboratory Animal Science, China Medical University, Shenyang, China; ^4^Key Laboratory of Jilin Province for Zoonosis Prevention and Control, Institute of Military Veterinary, Academy of Military Medical Sciences, Changchun, China

## Abstract

Postoperative cognitive dysfunction (POCD) is a severe complication of cardiopulmonary bypass (CPB) and has common characteristics such as acute cognitive dysfunction, impaired memory, and inattention. Mesenchymal stem cells (MSCs) are multipotent cells that have therapeutic potentials mainly through paracrine action via secreting growth factors and cytokines. Exosomes are one of the important paracrine factors and have been reported as potential cell-free therapy for the treatment of autoimmune and central nervous system disorders. In this study, we examined exosomes derived from antler MSCs (AMSCs) of POCD rats after CPB and evaluated their potential regulatory mechanisms. AMSC-derived exosomes reduced neurological damage and brain damage and prevent apoptosis in CPB rats. Furthermore, AMSC-derived exosomes were found to reduce hippocampal neuronal apoptosis and the expression of TLR2, TLR4, MyD88, and NF-*κ*B in CPB rats. However, the above effects of AMSC-derived exosomes on CPB rats were abolished partially by toll-like receptor 2/4 (TLR2/TLR4) agonist (LPS-EB). In conclusion, AMSC-derived exosomes can improve cognitive function in CPB rats through inhibiting the TLR2/TLR4 signaling pathway.

## 1. Introduction

The mortality rate of patients caused by postoperative postcentral nervous system (CNS) complications increases after cardiopulmonary bypass (CPB) surgery [1, 2]. POCD is a severe complication of CPB characterized by acute cognitive dysfunction, impaired memory, and inattention [3, 4]. Its onset arises from systemic and hippocampal inflammation during clinical postsurgery [5, 6]. Although the relevance of POCD to cardiac surgery has been identified, little is known about the underlying mechanisms, and effective prevention and treatment approaches are urgently needed.

Toll-like receptors (TLRs) are a group of highly conserved type I transmembrane proteins that can activate signal transduction pathways, induce inflammatory responses, and promote activation of antigen-presenting cells [7]. The TLR2/TLR4 signaling pathway plays a crucial role in various inflammatory diseases in the CNS. It has been found that endogenous activating substances released from damaged tissues after cerebral hemorrhage are recognized by TLR4; then, NF-*κ*B is activated and proinflammatory cytokines released, eventually leading to tissue damage [8]. Additionally, TLR2 is upregulated in ischemic brain tissue and injury-associated microglia, while cerebral ischemic injury is significantly reduced in TLR2 knockout mice [9].

MSCs are multipotent cells that can generate different cell types and form different tissues, such as bone, skin, and cartilage [10–14]. MSCs also have broad applications in the field of translational medicine such as tissue repair and regeneration, but the mechanism is still unclear [15]. Recent studies have shown that the therapeutic functions of MSCs mainly depend on paracrine action rather than their own proliferation and differentiation on site [16, 17]. Exosomes are one of the important paracrine factors that have been identified and have been demonstrated by more and more experiments to play a key role in the various functions of MSCs. Exosomes are important carriers for exchanging cellular information of which characteristics are related to their parental cells [18, 19]. Recent reports demonstrated that exosomes protect against neuroinflammatory properties in the CNS [20]. MSC-derived exosomes can potentially serve as cell-free therapy for a tolerogenic immune response to treat autoimmune and CNS disorders [21]. Importantly, intranasally administrated exosomes were rapidly transported to the brains of mice and absorbed by microglia. Moreover, the numbers of activated inflammatory microglia were found to be profoundly reduced compared with the control group [20].

Deer antler is a male secondary sexual appendage whose constant growth depends on seasonal changes throughout life [22, 23]. It is known that the rapid growth of antlers is mainly achieved through the proliferation of cells residing in the reserve mesenchyme [24]. Cells from the reserve mesenchyme have been isolated, cultured, and partially characterized by several laboratories, defined as a type of MSCs [25, 26]. According to previous studies, antlers have a variety of biological activities, including anti-inflammatory, tissue healing, antitumor, and antioxidation [27–29]. We reasoned MSCs isolated from antlers may have similar beneficial functions. This study evaluated the role of exosomes released from AMSCs in treating postoperative cognitive dysfunction in rats after cardiopulmonary bypass.

In summary, this work described the protective effects and mechanisms of AMSC-derived exosomes on POCD rats, which will provide theoretical and experimental evidence for the treatments of POCD patients after CPB.

## 2. Materials and Methods

### 2.1. Isolation of Antler Mesenchymal Stem Cells

Fresh sika deer antlers were obtained under conditions approved by the local Animal Ethics Committee and in accordance with the protocols approved by the Institute Animal Care. Antlers were collected from anesthetized three-year-old sika deer stags in late spring. Mesenchyme is a part of the antler tip that was obtained by slices of tissues taken using sterilized surgical instruments. The obtained samples were digested with 0.075% collagenase type II (Sigma-Aldrich, USA) under gentle agitation for 45 min at 37°C and then centrifuged at 300× g for 10 min to obtain a stromal cell fraction. The pellets were filtered through a 70 mm nylon mesh and resuspended in PBS. The solution was layered onto histopaque-1077 (Sigma-Aldrich, USA) and centrifuged at 840× g for 10 min. The supernatant was discarded, and cell fraction was cultured overnight at 37°C, in Gibco Dulbecco's Modified Eagle Medium: Nutrient Mixture F-12 (DMEM/F-12) supplemented with 10% fetal bovine serum (FBS), 100 units/ml of penicillin, and 100 mg/ml of streptomycin. AMSCs from passages 3-5 were used for further research.

### 2.2. Isolation and Purification of AMSC-Derived Exosomes

AMSCs were cultured in DMEM/F12 supplemented with 10% exosome-free FBS. After 48 h, exosomes were isolated from AMSC supernatant by an ultracentrifugation method according to the protocol of Thery et al. [30]. After ultracentrifuging at 120,000× g for 1.5 h, the exosomes were carefully resuspended in PBS and used immediately or stored at -80°C. The characteristic surface marker proteins (CD9, CD63, and Calnexin) of exosomes were analyzed by Western blot. The morphologies of exosomes were observed with a transmission electron microscope (TEM) as previously described. Flow Nano Analyzer model type N30 (Nano FCM Inc., Xiamen, China) that allows single exosome detection was used to determine the size distribution and granular concentration of AMSC-derived exosomes [31].

### 2.3. Transmission Electron Microscopy

The exosomes were loaded onto a formvar-coated grid and negatively stained with a neutral 1% aqueous phosphotungstic acid solution. Electron microscopic images were captured and analyzed by a transmission electron microscope (TEM, HT7700, 80 kV, Hitachi, Tokyo, Japan).

### 2.4. Laboratory Animals and Grouping

Forty male SPF SD rats weighing 350-450 g were provided by the Laboratory Animal Department of China Medical University (Rodent use license: SYXK 20180008; Rodent production license: SCXK 20180004). This experiment was approved by the Experimental Animal Ethics Committee of China Medical University (Approval number CMU2018170).

The rats were randomly divided into four groups: sham operation group (sham group), CPB surgery group (CPB group), exosome+CPB group (EXO group), and exosome+CPB group+TLR2/TLR4 agonist group (TLR group) with 10 animals in each group. Except for the sham group, all other groups were prepared using the POCD model by the CPB method. In the EXO group, the extracted AMSC-derived exosomes were administered 2.1 mg/kg peritoneal cavity 30 min before flow. In the TLR group, 2.5 mg/kg of TLR2/TLR4 agonist (LPS-EB) was injected into the tail vein 30 min before flow. After obtaining the data of water maze test, the rats were anesthetized and placed in a tube, 5 ml of blood was taken through the right internal vein, and the serum was separated by centrifugation and stored at -80°C. The bilateral hippocampus was taken immediately, and a part of the sample was stored in -80°C, while the other in formalin.

### 2.5. Preparation of the CPB Model

The animals were made to fast for 6 h before surgery and anesthetized by intraperitoneal injection of sodium (30 mg/kg). After the disappearance of righting reflex, the rats were tied to the operating table and intubated on the trachea. The small animal ventilator was connected, and electrocardiogram, blood oxygenation saturation, and body temperature were monitored continuously. The left femoral artery was placed in a catheter and connected to an arterial kit to measure real-time arterial pressure. The left femoral vein was placed in the needle and the colloid continuously pumped. The tail artery was placed in a needle tube, and the CPB was infused to be prepared for use. Then, the right internal jugular vein was drained. A total of 15 ml of circulating prefilled liquid was prepared, including 6 ml of hydroxyethyl starch, 1 ml of heparin (the content is 250 IU/kg), 1 ml of 5% sodium bicarbonate, 1 ml of 20% mannitol, and 1 ml of furosemide. Six ml of prefilled liquid was added to connect and fix each pipe. During the CPB process, the flow rate was adjusted in real time, and the blood volume in the blood reservoir was maintained at 1 to 2 ml. The flow rate of extracorporeal circulation was not less than 80 ml/kg/min. After 1 h of the CPB process, mechanical ventilation was resumed. The hematocrit (Hct) reached above 0.25, the flow rate was gradually decreased, and the machine was stopped. The tubes in the heart were removed one at a time and mechanical ventilation was continued. The remaining blood was slowly infused in the reservoir, and the tracheal intubation was withdrawn after the rats resumed spontaneous breathing.

### 2.6. Water Maze

24 h after CPB, the water maze test was performed for 7 days, including hidden platform test and space exploration test. In the hidden platform test, the rats were placed from any quadrant into the water facing the pool wall and had to swim for 90 s to find the hidden platform. The time to find the hidden platform is the period for escaping the incubation. If the rats were unable to find the platform within 90 s, they were directed to the platform with a recording score of 90 s. The experiment was conducted for 5 days, and the first 4 days was used as acquired training. The score of 90 s was eliminated, and the test scores on day 5 were used as the spatial learning memory scores. In space exploration testing, after 24 h of the hidden platform test, the platform was removed. The rats were placed inside the water at the same water inlet point, and the swimming path of the rats was recorded within 60 s. Furthermore, the swimming distance and residence time of the target quadrant and the number of crossings of the original station were recorded. The moving trajectories of the rats were recorded, and the information processing was performed using the Morris water-maze video analysis system.

### 2.7. The Score for Neurological Function Score

After 1 day, 3 days, and 7 days of CPB bypass, the neurological function of the experimental animals was measured using the Garcia score scale (Table 1).

### 2.8. H&E Staining

Tissue samples fixed in formalin were placed in alcohol at a concentration of 70%, 80%, 90%, 95%, and 100%. Xylene was transparent and dipped in wax blocks. Then, the wax blocks were cut into 4 *μ*m sections with a microtome and then dewaxed. The sections were stained with hematoxylin for 5 min, washed with PBS, and differentiated with 1% hydrochloric acid alcohol and eosin dye solution for 30 s. After the gradient alcohol dehydration, transparent treatment, and neutral gum seal, the pathological changes in each group were observed under light microscopy.

### 2.9. TUNEL Staining

The *in situ* cell death assay kit (Roche Diagnostics, Mannheim, Germany) was used according to the manufacturer's instructions. Briefly, 5 *μ*mol/l sections of paraffin-embedded hippocampal tissue were deparaffinized, permeabilized, and blocked. Sections were treated with 50 *μ*l TUNEL reaction solution and incubated in a humid dark box at 37°C for 60 min. Subsequently, 50 *μ*l of the streptavidin-HRP working solution was added into the sections and incubated again in the cassette for 30 min. Fluorescent staining used DAPI to stain nuclei, followed by conventional dehydration, decolorization, and fixation. The sections were examined with a microscope, and the images were taken. For each captured image, the total number of nuclei and TUNEL-positive cell nuclei was counted, and the proportion of apoptosis was calculated.

### 2.10. ELISA Test

Inflammatory factors IL-1*β* (CSB-E08055r, CUSABIO, China), IL-6 (SEA079Ra, USCN, China), TNF-*α* (SEA133Si, USCN, China), IL-10 (SEA056Ra, USCN, China), oxidative stress index SOD (SES134Hu, USCN, China), MDA (CEA597Ge, USCN, China), GSH (CEA294Ge, USCN, China), NO (IS100, USCN, China), and brain injury markers S100-*β* (SEA567Ra, USCN, China) and NSE (SEA537 Ra, USCN, China) in rat serum were detected using different ELISA kits, and the operation steps were carried out according to the manufacturer's instructions.

### 2.11. Immunofluorescence Assay

After dewaxing, the wax pieces of hippocampal tissue were immersed in a 3% hydrogen peroxide solution for 15 min, washed with PBS three times, and subjected to antigen retrieval by a 0.1 M sodium citrate solution. It was blocked in goat serum and incubated at 37°C for 30 min. Then, the wax pieces of hippocampal tissue were incubated with primary antibodies (anti-TLR2, Abcam, ab16894; anti-TLR4, Abcam, ab22048; anti-MyD88, Abcam, ab28763; and anti-NF-*κ*B, Abcam, ab16502; Cambridge, UK) overnight at 4°C, washed with PBS three times, incubated with the appropriate secondary antibodies labeled with fluorescence for 30 min at 37°C, and then washed with PBS three times. Finally, the sections were stained with DAPI and incubated for 10 min at room temperature and then washed with PBS three times, sealed with a neutral resin, and observed under a fluorescence microscope.

### 2.12. Western Blot

After homogenization of the exosomes and hippocampus, precooled RIPA (Thermo, 89900, USA) lysate was added and lysed on ice for 30 min. The supernatant was collected, and the concentration was measured by a BCA protein quantification kit (Thermo, 23225, USA). Briefly, 40 *μ*g of proteins in the supernatant was separated by SDS-PAGE and transferred onto a PVDF membrane. CD9 (Abcam, ab92726, UK), CD63 (Abcam, ab59479,), and Calnexin (Abcam, ab22595,) primary antibodies were added to the exosomal tissue, and TLR2, TLR4, MyD88, and NF-*κ*B were added to the hippocampus. Bcl 2 (Abcam, ab59348), Bax (Abcam, ab32503), pro-caspase-3 (Abcam, ab32150), and cleaved caspase-3 (Abcam, ab49822) primary antibodies were added onto the PVDF membrane and incubated overnight at 4°C. After washing with PBS, secondary antibodies were added and incubated at room temperature for 2 h. The ECL luminescence kit (Thermo Scientific, USA) was used to develop the color. Images were obtained by the Azure c600 Western Blot Imaging System (Azure, USA), and the gray values were read by Quantity One software.

### 2.13. PCR

Frozen brain tissue lysis was prepared by Trizol (Invitrogen, 15596026, USA). Total RNA of tissues and cells were extracted according to the Trizol reagent operating instructions. After the first-strand cDNA (Thermo, K1622, USA) was synthesized by reverse transcription, TLR2, TLR4, MyD88, and NF-*κ*B reactions were detected by qRT-PCR (Qiagen, 204057, Germany). The primer sequences are shown in Table 2, which were synthesized by Shanghai Shenggong Biological Co., Ltd. The reaction conditions were predenaturation: 95°C for 30 s; PCR reaction: 95°C for 5 s and 60°C for 20 s, 40 cycles; and melting curve analysis: 95°C for 1 s, 65°C for 15 s, and 95°C for 5 s. After the reaction was completed, the amplification curve and the melting curve were confirmed.

### 2.14. Statistical Analysis

Results were analyzed using SPSS 19.0 (SPSS, Chicago, IL, USA) software. The measurement data were expressed as mean ± standard deviation (*x* ± *s*). The *t*-test was used for comparison between two groups, and the difference was statistically significant when *p* < 0.05.

## 3. Results

### 3.1. Morphological and Phenotypic Identification of Collected Exosomes

**Figure 1:**
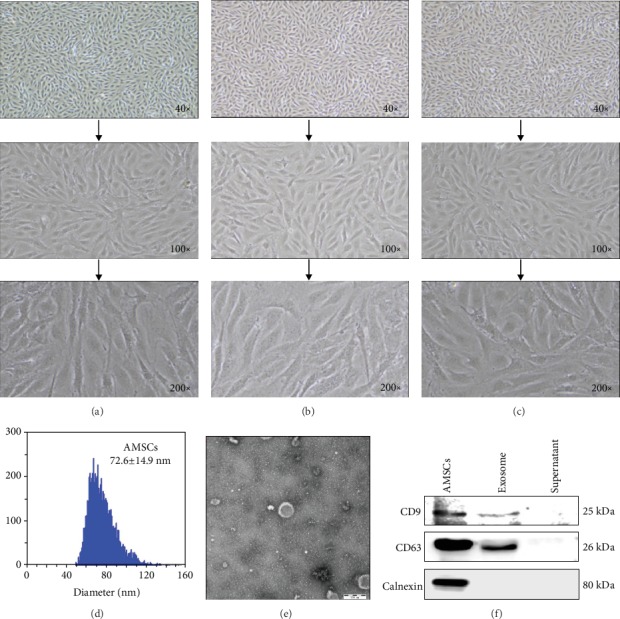
Morphological and phenotype identification of collected exosomes. (a–c) Representative bright-field microscopy image of AMSCs. (d) Size distribution of exosomes determined by Flow Nano Analyzer. (e) Representative electron microscopy image of AMSC-derived exosomes. (F) Representative Exo-Check antibody of isolated exosomes detected by Western blot.

AMSCs were subcultured using 0.25% trypsin-EDTA solution *in vitro*. After 3 days of cultivation under normal conditions, AMSCs were found to be fusiform, triangular, or polygonal in shape, and the cytoplasm was transparent (Figure 1(a)). The fused cells were arranged in a monolayer (Figure 1(b)) after 7 days of cultivation and fusion. In the 5^th^ generation, the cells were evenly and closely arranged, becoming fusiform or triangular. After the 5^th^ generation, the morphologies of certain cells changed, including flattening of cells, the acceleration of cellular processes, and the inconsistency of cells (Figure 1(c)). The range and size of the exosomes were measured using the Flow Nano Analyzer model type N30 (Nano FCM Inc., Xiamen, China). The results demonstrated that the diameters of the particles were within the range of 50-160 nm, with an average of 72.6 nm (Figure 1(d)). The morphologies of the exosomes were directly observed through TEM. The particles were observed as round-shaped vesicles with a bilayer membrane structure and with a diameter of approximately 50-100 nm (Figure 1(e)). The protein markers CD9, CD63, and Calnexin of the exosomes were identified by Western blot (Figure 1(f)). The above results indicate that the exosomes have been successfully isolated from AMSCs.

### 3.2. AMSC-Derived Exosomes Alleviated Neurological Damage in CPB Rats

**Figure 2:**
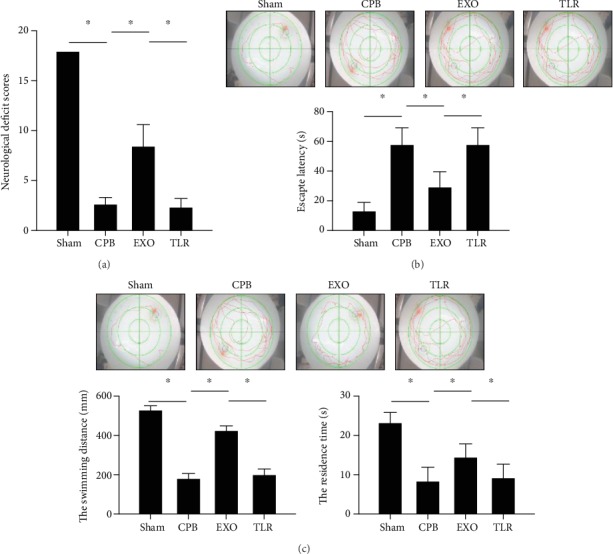
AMSC-derived exosomes alleviated neurological damage in CPB rats. SPF SD male rats were randomly divided into four groups including the sham operation group (sham group); CPB surgery group (CPB group); exosome+CPB group (EXO group); and exosome+CPB group+TLR2/TLR4 agonist group (TLR group) with 10 rats in each group. (a) The neurological function scores in each group. (b) The escape latency in each group. (c) The swimming distance and residence time in each group; ^∗^*p* < 0.05 (*n* = 10).

CPB can lead to neurological and mental disorders represented by POCD. CPB rats were injected with AMSC-derived exosomes, and the changes in neurological function scores and spatial learning and memory abilities were observed. The neurological function scores of the CPB group were 2.7 ± 0.6, which were significantly lower than those of the sham group (*p* < 0.05) (Figure 2(a)). After the administration of the exosomes, the neurological function scores increased to 8.5 ± 2.1 compared with the CPB group (*p* < 0.05). The hidden platform training and space exploration experiments were performed to test the cognitive function of rats. In Figure 2(b), compared to the sham group, the latency for finding the platform in the CPB group was obviously longer (*p* < 0.05). Compared with the CPB group, the latency period in the EXO group was significantly shorter (*p* < 0.05). The latency period in the TLR group was similar to that of the CPB group (*p* > 0.05). In the space exploration experiment (Figure 2(c)), the swimming distance and residence time of the target quadrant were significantly reduced in the CPB group compared with the sham group (*p* < 0.05). The swimming distance and residence time of rats in the EXO group were significantly longer than those in the CPB group (*p* < 0.05). Furthermore, the swimming distance and residence time were seen to improve by intervention of TLR2/4 signaling agonist (*p* < 0.05). The above results suggest that AMSC-derived exosomes can alleviate neurological damage in CPB rats.

### 3.3. AMSC-Derived Exosomes Prevent Brain Damage in CPB Rats

**Figure 3:**
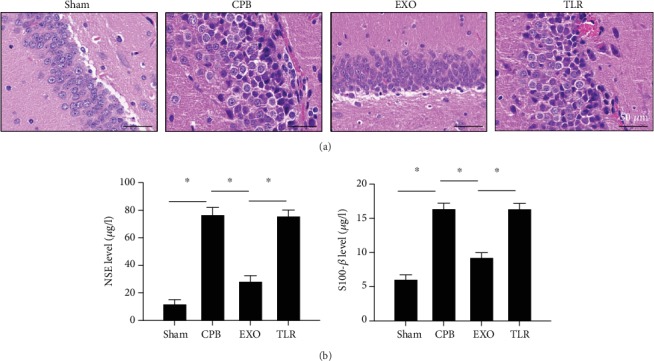
AMSC-derived exosomes prevent brain damage in CPB rats. SPF SD male rats were randomly divided into four groups including the sham operation group (sham group); CPB surgery group (CPB group); exosome+CPB group (EXO group); and exosome+CPB group+TLR2/TLR4 agonist group (TLR group) with 10 rats in each group. (a) H&E staining revealed the damage of hippocampus tissues in each group (scale bar = 50 *μ*m). (b) Brain injury markers (NSE and S100-*β*) in serum in each group; ^∗^*p* < 0.05 (*n* = 10).

Histopathological changes of brain tissue were observed through H&E staining. As shown in Figure 3(a), the hippocampus of CPB rats was found to be seriously damaged, the cells dispersed, and the intercellular space gradually expanded. In addition, the proliferation of cell and vascular tissue increased and the tissue structure and arrangement were disordered. Compared with the CPB group, in the EXO group, the cells were neatly arranged and the cell band was incomplete. The damage condition of the TLR group was similar to that in the CPB group. The cells were sparsely distributed, and the cytoplasmic vacuoles were gradually increased. H&E staining revealed that the hippocampus tissues of CPB rats were seriously damaged, and the intervention of AMSC-derived exosomes could prevent hippocampus tissue damages. Moreover, TLR2/TLR4 agonist partially reversed the prevented effect of AMSC-derived exosomes on CPB-induced brain damage in CPB rats. To verify the extent of hippocampal damage in CPB rats, ELISA was used to detect markers of brain injury (Figure 3(b)). The results showed that serum NSE and S100-*β* concentrations in the CPB group had significantly increased (vs. sham, *p* < 0.05). The markers in the EXO group were significantly lower than those in the CPB group (*p* < 0.05) while those in the TLR group were similar to those in the CPB group (*p* > 0.05). These results suggested that AMSC-derived exosomes can prevent brain damage in CPB rats.

### 3.4. AMSC-Derived Exosomes Inhibited Inflammation and Oxidative Stress in CPB Rats

**Figure 4:**
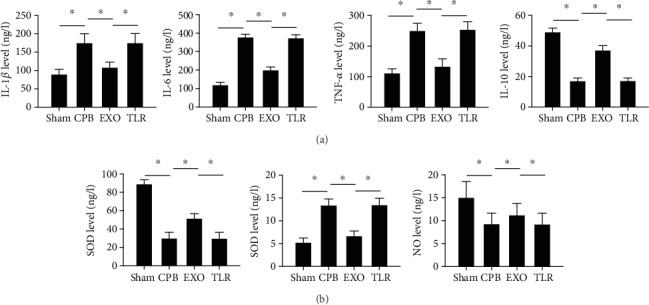
AMSC-derived exosomes inhibited inflammation and oxidative stress in CPB rats. SPF SD male rats were randomly divided into four groups including the sham operation group (sham group); CPB surgery group (CPB group); exosome+CPB group (EXO group); and exosome+CPB group+TLR2/TLR4 agonist group (TLR group) with 10 rats in each group. (a) The concentrations of inflammatory factors (IL-1*β*, IL-6, TNF-*α*, and IL-10) in serum in each group. (b) The levels of oxidative stress factors (SOD, MDA, and NO) in serum in each group; ^∗^*p* < 0.05 (*n* = 10).

Inflammatory factors and oxidative stress factors in rat serum were detected by ELISA. The results are shown in Figure 4. Compared with the sham group, the concentrations of IL-1*β*, IL-6, and TNF-*α* increased while those of IL-10 decreased significantly in the CPB group (*p* < 0.05), indicating that CPB can lead to an increase in inflammatory response. In the EXO group, the concentrations of IL-1*β*, IL-6, and TNF-*α* were significantly reduced, and IL-10 was significantly increased (vs. CPB, *p* < 0.05) (Figure 4(a)). These results suggest that AMSC-derived exosomes can effectively inhibit the inflammatory response in CPB rats. Similarly, the SOD and NO levels in the CPB group were significantly lower than those in the sham group, and the MDA content had increased significantly (*p* < 0.05). In the EXO group, the SOD and NO levels were significantly higher than those in the CPB group, and the MDA content had reduced significantly (*p* < 0.05) (Figure 4(b)). These results suggested that AMSC-derived exosomes can inhibit the oxidative stress response in CPB rats.

### 3.5. AMSC-Derived Exosomes Prevented Neuronal Apoptosis in CPB Rats

**Figure 5:**
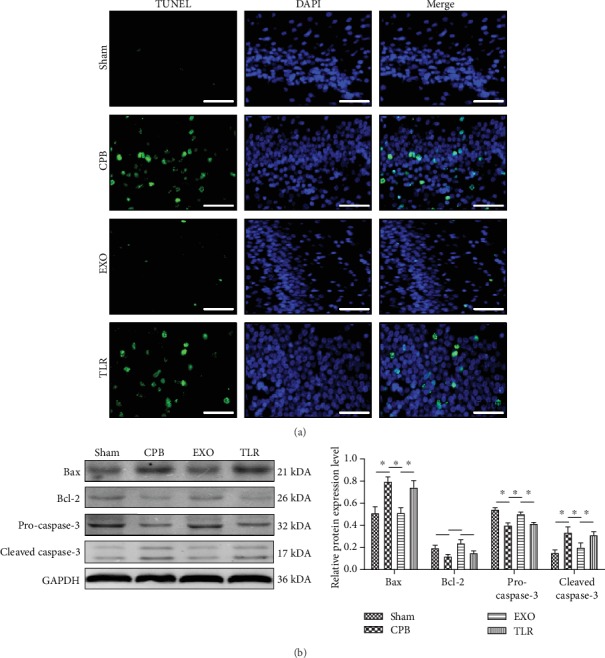
AMSC-derived exosomes prevented neuronal apoptosis in CPB rats. SPF SD male rats were randomly divided into four groups including the sham operation group (sham group); CPB surgery group (CPB group); exosome+CPB group (EXO group); and exosome+CPB group+TLR2/TLR4 agonist group (TLR group) with 10 rats in each group. (a) Neuronal apoptosis in brain tissue in each group was detected by TUNEL (scale bar = 50 *μ*m). (b) Apoptosis-related protein expression in hippocampus tissue in each group was determined by Western blot; ^∗^*p* < 0.05 (*n* = 10).

POCD can cause the apoptosis of brain neurons. Neuronal apoptosis in the brain tissue was detected by TUNEL (Figure 5(a)), and the expressions of apoptosis-related proteins were measured by Western blot (Figure 5(b)). TUNEL results show that the number of positive cells in the CPB group increased significantly (vs. sham, *p* < 0.05). Furthermore, the positive cells of the hippocampus in the EXO group were significantly lower than that those in the CPB group (*p* < 0.05), while the number of positive cells in the TLR group had significantly increased compared with that in the EXO group (*p* < 0.05). These results indicated that AMSC-derived exosomes have a protective effect on the neuronal apoptosis in CPB rats. Similarly, as shown in Figure 5(b), Bcl-2 expression was seen to significantly decrease in the CPB group and significantly increase in the Bax (vs. sham, *p* < 0.05). The expression of Bcl-2 in the EXO group had significantly increased, while in Bax significantly decreased (vs. CPB, *p* < 0.05). However, Bcl-2 expression was found to significantly decrease in the TLR group and significantly increase in the Bax (vs. EXO, *p* < 0.05). In addition, the expression of pro-caspase-3 in the CPB group significantly decreased, and the expression of cleaved caspase-3 significantly increased (vs. sham, *p* < 0.05). The expression of pro-caspase-3 significantly increased in the EXO group, while the expression of cleaved caspase-3 significantly decreased (vs. CPB, *p* < 0.05). Compared to the EXO group, the expression of pro-caspase-3 in the TLR group significantly decreased, while cleaved caspase-3 level significantly increased (*p* < 0.05). These results suggest that AMSC-derived exosomes can inhibit neuronal apoptosis and prevent neuronal degeneration in CPB rats.

### 3.6. AMSC-Derived Exosomes Improved CPB-Induced POCD in Rats via the TLR2/TLR4 Signaling Pathway

**Figure 6:**
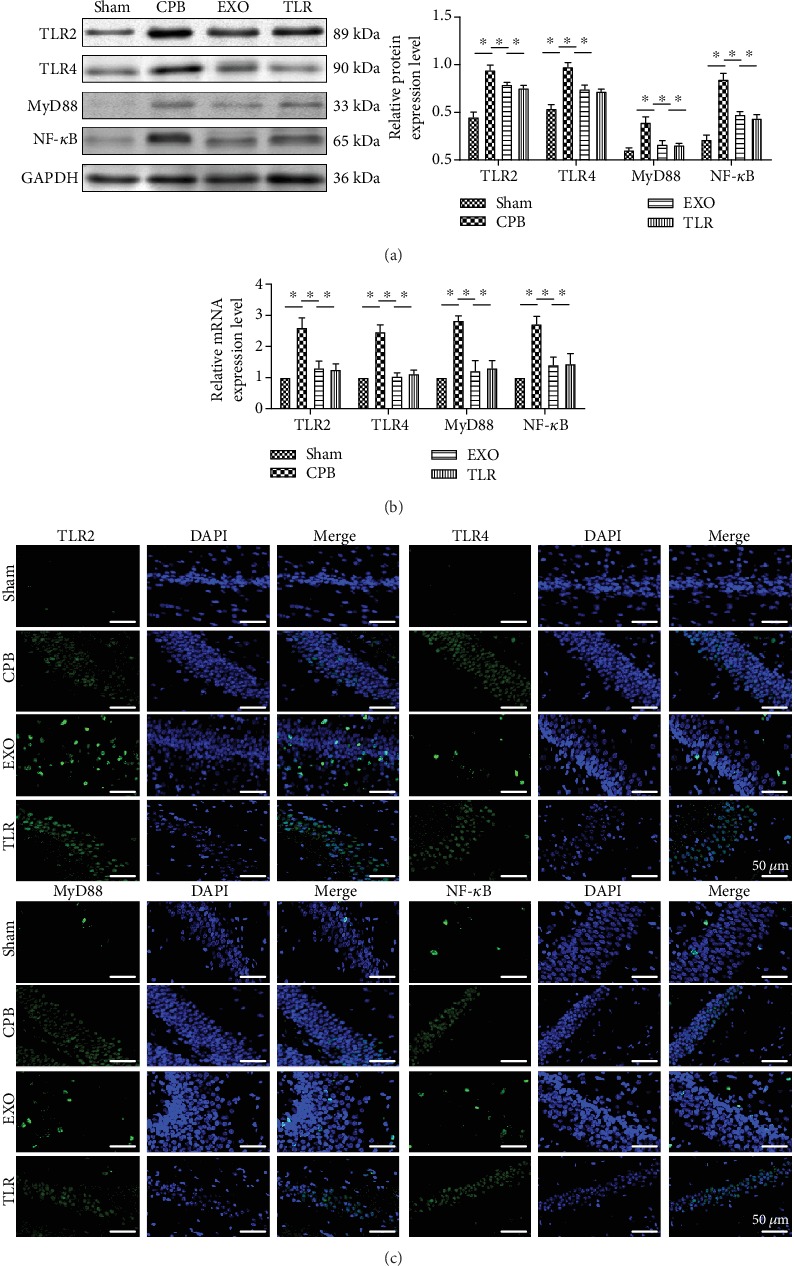
AMSC-derived exosomes improved CPB-induced POCD in rats via the TLR2/TLR4 signaling pathway. SPF SD male rats were randomly divided into four groups including the sham operation group (sham group); CPB surgery group (CPB group); exosome+CPB group (EXO group); and exosome+CPB group+TLR2/TLR4 agonist group (TLR group) with 10 rats in each group. (a, b) TLR2, TLR4, MyD88, and NF-*κ*B expression in hippocampus tissue in each group was determined by Western blot and qRT-PCR. (c) The immunofluorescence image of TLR2, TLR4, MyD88, and NF-*κ*B in hippocampus tissue in each group (scale bar = 50 *μ*m); ^∗^*p* < 0.05 (*n* = 10).

Toll-like receptors play an important role in the inflammatory and immune responses of CPB rats. Therefore, the levels of TLR2, TLR4, MyD88, and NF-*κ*B in the hippocampus of rats were detected by Western blot, qRT-PCR, and immunofluorescence. As shown in Figures 6(a) and 6(b), the mRNA and protein expressions of TLR2, TLR4, MyD88, and NF-*κ*B in the CPB group were higher than those in the sham group (*p* < 0.05). Compared with the CPB group, the levels of TLR2, TLR4, MyD88, and NF-*κ*B in the EXO group were much lower (*p* < 0.05), suggesting that AMSC-derived exosomes can reverse the expression of TLR2/TLR4 signaling pathway-related proteins in the CPB rats. Meanwhile, after activation of the TLR2/TLR4 signaling pathway, the exosomes cannot decrease IL-1*β*, IL-6, TNF-*α*, and MAD level and also cannot increase the levels of IL-10, SOD, and NO (Figures 4(a) and 4(b)). Furthermore, these results were confirmed by immunofluorescence (Figure 6(c)). It can be seen that AMSC-derived exosomes could alleviate POCD in CPB rats through the TLR2/TLR4 signaling pathway.

## 4. Discussion

Our study revealed that AMSC-derived exosomes have neuroprotective effects on rats after cardiopulmonary bypass. *In vivo*, we showed that intraperitoneal administration of AMSC-derived exosomes can reduce inflammatory response and oxidative stress and prevent neuronal apoptosis and brain damage, thereby hindering the development of POCD in CPB rats.

The application of extracorporeal circulation technology in clinical practice is expanding, and POCD is becoming a serious complication in CPB surgery [32]. This problem is directly related to the success of surgical treatment and seriously perplexes the development of cardiac surgery. The symptoms of POCD are closely correlated with neuroinflammation, which is similarly to Alzheimer's disease (AD) [33]. During the growth of deer antler, the proliferation of mesenchymal cells determines the growth rate of antler [24]. Mesenchymal cells are regarded as the major cell source of velvet growth and can differentiate into fibroblasts, chondrocytes, and osteocytes during velvet growth [25, 26]. In addition to the multipotent differentiation potential of MSCs, several paracrine factors of MSCs have been discovered that act on cell migration, antioxidation, antiapoptosis, angiogenesis, and immunomodulation via regulating local cellular responses. MSC-derived exosomes play an important role in neuroinflammation, neurogenic niches and neurogenesis, and therapeutic strategy of neurological diseases. In our study, AMSCs and their exosomes were successfully isolated and identified (Figures 1(a)–1(f)). The POCD model of CPB rats was established to investigate the protective effect of AMSC-derived exosomes. The neurological function score and water-maze test were performed on the rats to confirm that AMSC-derived exosomes significantly increased neurological function scores, swimming distance, and residence time and have shorter latency period compared to the CPB group (Figures 2(a)–2(c)). Neuron-specific enolase (NSE) and S100 calcium-binding protein B (S100-*β*) are considered the specific and sensitive biochemical markers of CNS damage [34]. As shown in Figure 3(a), AMSC-derived exosomes prevented the damage of hippocampus tissues and significantly reduced the serum level of NSE and S100-*β*.

Inflammation is considered a major characteristic of several pathological conditions. Inflammation amplifiers play key roles in the development and progression of various human diseases, including autoimmune, neurodegenerative, and other inflammatory diseases [35]. Activation of the immune system by surgery was identified as a strong stimulation of the immune cell infiltration through a disrupted blood-brain barrier (BBB), such as macrophages and neutrophils. The resulting cerebral inflammation can result in cognitive decline [36]. As shown by a recent study, exosomes derived from human Wharton's jelly mesenchymal stem cells (WJ-MSCs) have been shown to have anti-inflammatory effects on microglia in perinatal brain injury [37]. Similar to this result, exosomes derived from adipose stem cells can inhibit microglial activation and prevent neuroinflammation by inhibiting the NF-*κ*B and MAPK pathways [38]. In our study, plasma IL-1*β*, IL-6, and TNF-*α* levels were seen to significantly increase in the CPB group, and this increase was suppressed by AMSC-derived exosomal supplementation (Figure 4(a)). IL-10, a key molecule in inhibiting proinflammatory cytokine production, was decreased in the EXO group, and the effect was reversed by AMSC-derived exosomal supplementation (Figure 4(a)). Oxidative stress plays a key role in the development and progression of neuroinflammation due to its activation in the CNS immune system, which results in a central inflammatory response that stimulates the production of several inflammatory mediators, such as more reactive species, chemokines and cytokines, microglial activation, and the infiltration of innate and adaptive immune cells from the periphery [39]. In the present study, exosomes from human umbilical cord MSCs alleviate the oxidative injury via oxalate+COM in HK-2 cells [40]. Associated with this, we have demonstrated that POCD can induce an increase in MDA levels, while SOD and NO levels can be reversed by AMSC-derived exosomes (Figure 4(b)). Data from a series of studies have reported that when POCD occurs, apoptosis is observed in the hippocampus, which serves an important role in the occurrence and development of POCD [41]. In our studies, the results of TUNEL showed a significant increase in the number of positive cells in the CPB group, and this increase was seen to be suppressed by AMSC-derived exosomal supplementation (Figure 5(a)). Furthermore, in the expression of Bcl-2, pro-caspase-3 significantly decreased, and in the expression of Bax, cleaved-caspase-3 significantly increased in the CPB group; these effects were partially abolished by AMSC-derived exosomes (Figure 5(b)).

TLRs are well-known crucial pattern recognition receptors (PRRs) that are involved in the recognition of pathogen-associated molecular patterns (PAMPs) [42]. Later studies have shown that TLRs are responsible for the pathogenesis of neuroinflammation under diverse conditions. For example, microglia lacking the expression of TLR2, TLR4, and CD14 are unable to cause a proinflammatory immune response, which can decrease the expression of proinflammatory cytokines (e.g., TNF-*α*, IL-1*α*, IL-6, and IL-8) [43, 44]. Additionally, it has been reported that WJ-MSC-derived exosomes inhibit LPS-induced TLR4/CD14 signaling in microglial cell [39]. Our study revealed that AMSC-derived exosomes improved CPB-induced POCD in rats via the TLR2/TLR4 signaling pathway. As shown in Figure 6, mRNA and protein expressions of TLR2, TLR4, MyD88, and NF-*κ*B significantly increased in the CPB group, and this increase was inhibited by AMSC-derived exosomal supplementation. Nevertheless, the TLR2/TLR4 agonist (LPS-EB) partially reversed the inhibitory effect of AMSC-derived exosomes on CPB and induced the expressions of TLR2, TLR4, MyD88, and NF-*κ*B. Moreover, TLR2/TLR4 agonists were also seen to partially reverse the effects of AMSC-derived exosomes on the CPB group (Figures 2–5).

In summary, AMSC-derived exosomes can reduce POCD brain damage, inflammation, and oxidative stress and prevent cell apoptosis in CPB rats. This inhibition is associated with the TLR2/TLR4 signaling pathway.

## 5. Conclusions

This study demonstrated that AMSC-derived exosomes can help improve cognitive function in rats subjected to CPB, which is associated with the TLR2/TLR4 signaling pathway. We identified exosomes derived from AMSCs and investigated their ability to alleviate neurological damage and brain damage and prevent neuronal apoptosis in CPB rats through inhibiting the TLR2/TLR4 signaling pathway. These results suggested the protective effects and mechanism of AMSC-derived exosomes on POCD rats. Therefore, our studies provided theoretical and experimental evidence for the prevention, occurrence, development, and prognosis of cognitive impairment after cardiopulmonary bypass.

## Figures and Tables

**Table 1:** The Garcia score scale.

Test content	0	1	2	3
5 min free activity in the cage	No activity	Almost inactive	Active, while the range for activities could not reach 3 sides in the cage	The range for activities could reach at least 3 sides in the cage
Symmetry of limb movements	Inactive in the left limb	Left limbs can be slightly active	Left limbs can move slowly	Bilateral limbs could be active symmetrically
Symmetry of the forelimbs	Inactive in the left limb	The left limb can only be slightly stretched	The left limb is less active and stretched than the right	The bilateral forelimbs can be stretched symmetrically
Climbing in a metal cage	Nothing	Unable to climb	The left side is slightly weak	Able to climb
The response of touching the bilateral trunk	Nothing	No response in the left side	Left side reacts slightly	Responds symmetrically
Tactile response	Nothing	No response in the left side	Left side reacts slightly	Responds symmetrically

**Table 2:** qRT-PCR using gene primers.

Gene	Primer
	(5′→3′)
TLR2	Forward: CGGAGGTCATCTCAGGAAGG
	Reverse: CGATCAGCAGAGTGGCAATAG
TLR4	Forward: AAGGGCTTCTACTCAGAG
	Reverse: AGGACCCACATGGGCACT
MyD88	Forward: GTAGCCAGCCTCTGAAAC
	Reverse: AGCCAGGATGATGTCTAC
NF-*κ*B	Forward: TTTCAAAAGTGGCATTGCTT
	Reverse: TTAAGCTGTAAAATCACA
GAPDH	Forward: GTCATCAACGGGAAACC
	Reverse: CATGGAGAAGGCTGGGG

## Data Availability

The datasets used and/or analyzed during the current study are available from the corresponding author on reasonable request.

## References

[1] Butala B., Busada M., Cormican D. (2018). Malignant hyperthermia: review of diagnosis and treatment during cardiac surgery with cardiopulmonary bypass. *Journal of Cardiothoracic and Vascular Anesthesia*.

[2] Ramaiah R., Lam A. M. (2009). Postoperative cognitive dysfunction in the elderly. *Anesthesiology Clinics*.

[3] Krenk L., Rasmussen L. S., Kehlet H. (2010). New insights into the pathophysiology of postoperative cognitive dysfunction. *Acta Anaesthesiologica Scandinavica*.

[4] Vacas S., Degos V., Feng X., Maze M. (2013). The neuroinflammatory response of postoperative cognitive decline. *British Medical Bulletin*.

[5] Block M. L., Zecca L., Hong J. S. (2007). Microglia-mediated neurotoxicity: uncovering the molecular mechanisms. *Nature Reviews Neuroscience*.

[6] Prinz M., Priller J. (2017). The role of peripheral immune cells in the CNS in steady state and disease. *Nature Neuroscience*.

[7] Achek A., Yesudhas D., Choi S. (2016). Toll-like receptors: promising therapeutic targets for inflammatory diseases. *Archives of Pharmacal Research*.

[8] Liu D. L., Zhao L. X., Zhang S., du J. R. (2016). Peroxiredoxin 1-mediated activation of TLR4/NF-*κ*B pathway contributes to neuroinflammatory injury in intracerebral hemorrhage. *International Immunopharmacology*.

[9] Lehnardt S., Lehmann S., Kaul D. (2007). Toll-like receptor 2 mediates CNS injury in focal cerebral ischemia. *Journal of Neuroimmunology*.

[10] Phelps J., Sanati-Nezhad A., Ungrin M., Duncan N. A., Sen A. (2018). Bioprocessing of Mesenchymal Stem Cells and Their Derivatives: Toward Cell- Free Therapeutics. *Stem Cells International*.

[11] Harrell C. R., Markovic B. S., Fellabaum C., Arsenijevic A., Volarevic V. (2019). Mesenchymal stem cell-based therapy of osteoarthritis: Current knowledge and future perspectives. *Biomedicine & pharmacotherapy*.

[12] Jung S. N., Rhie J. W., Kwon H. (2010). In vivo cartilage formation using chondrogenic-differentiated human adipose-derived mesenchymal stem cells mixed with fibrin glue. *The Journal of Craniofacial Surgery*.

[13] Motegi S. I., Ishikawa O. (2017). Mesenchymal stem cells: the roles and functions in cutaneous wound healing and tumor growth. *Journal of Dermatological Science*.

[14] Wang W., Li P., Li W. (2017). Osteopontin activates mesenchymal stem cells to repair skin wound. *PLoS One*.

[15] Liang X., Ding Y., Zhang Y., Tse H. F., Lian Q. (2014). Paracrine mechanisms of mesenchymal stem cell-based therapy: current status and perspectives. *Cell Transplantation*.

[16] Han C., Sun X., Liu L. (2016). Exosomes and their therapeutic potentials of stem cells. *Stem Cells International*.

[17] Lai R. C., Yeo R. W., Lim S. K. (2015). Mesenchymal stem cell exosomes. *Seminars in Cell & Developmental Biology*.

[18] Kowal J., Tkach M., Thery C. (2014). Biogenesis and secretion of exosomes. *Current Opinion in Cell Biology*.

[19] Regev-Rudzki N., Wilson D. W., Carvalho T. G. (2013). Cell-cell communication between malaria-infected red blood cells via exosome-like vesicles. *Cell*.

[20] Zhuang X., Xiang X., Grizzle W. (2012). Corrigendum: Treatment of brain inflammatory diseases by delivering exosome encapsulated anti-inflammatory drugs from the nasal region to the brain. *Molecular Therapy*.

[21] Constantinescu C. S., Farooqi N., O'Brien K., Gran B. (2011). Experimental autoimmune encephalomyelitis (EAE) as a model for multiple sclerosis (MS). *British Journal of Pharmacology*.

[22] Li C., Harper A., Puddick J., Wang W., McMahon C. (2012). Proteomes and signalling pathways of antler stem cells. *PLoS One*.

[23] Cegielski M., Izykowska I., Chmielewska M. (2013). Characteristics of MIC-1 antlerogenic stem cells and their effect on hair growth in rabbits. *In vivo*.

[24] Li C., Suttie J. M., Clark D. E. (2004). Morphological observation of antler regeneration in red deer (Cervus elaphus). *Journal of Morphology*.

[25] Rolf H. J., Kierdorf U., Kierdorf H. (2008). Localization and characterization of STRO-1 cells in the deer pedicle and regenerating antler. *PLoS One*.

[26] Seo M. S., Park S. B., Choi S. W., Kim J. J., Kim H. S., Kang K. S. (2014). Isolation and characterization of antler-derived multipotent stem cells. *Cell Transplantation*.

[27] Lee S. H., Yang H. W., Ding Y. (2015). Anti-inflammatory effects of enzymatic hydrolysates of velvet antler in raw 264.7 cells in vitro and zebrafish model. *EXCLI Journal*.

[28] Mikler J. R., Theoret C. L., High J. C. (2004). Effects of topical elk velvet antler on cutaneous wound healing in streptozotocin-induced diabetic rats. *Journal of alternative and complementary medicine*.

[29] Tang Y., Fan M., Choi Y. J. (2019). Sika deer (Cervus nippon) velvet antler extract attenuates prostate cancer in xenograft model. *Bioscience, Biotechnology, and Biochemistry*.

[30] Théry C., Amigorena S., Raposo G., Clayton A. (2006). Isolation and characterization of exosomes from cell culture supernatants and biological fluids. *Current protocols in cell biology*.

[31] Tian Y., Ma L., Gong M. (2018). Protein profiling and sizing of extracellular vesicles from colorectal cancer patients via flow cytometry. *ACS Nano*.

[32] Pappa M., Theodosiadis N., Tsounis A., Sarafis P. (2017). Pathogenesis and treatment of post-operative cognitive dysfunction. *Electronic Physician*.

[33] Fodale V., Santamaria L. B., Schifilliti D., Mandal P. K. (2010). Anaesthetics and postoperative cognitive dysfunction: a pathological mechanism mimicking Alzheimer's disease. *Anaesthesia*.

[34] Luo X., Zheng X., Huang H. (2016). Protective effects of dexmedetomidine on brain function of glioma patients undergoing craniotomy resection and its underlying mechanism. *Clinical Neurology and Neurosurgery*.

[35] Atsumi T., Singh R., Sabharwal L. (2014). Inflammation amplifier, a new paradigm in cancer biology. *Cancer Research*.

[36] Solas M., Milagro F. I., Ramírez M. J., Martínez J. A. (2017). Inflammation and gut-brain axis link obesity to cognitive dysfunction: plausible pharmacological interventions. *Current Opinion in Pharmacology*.

[37] Thomi G., Surbek D., Haesler V., Joerger-Messerli M., Schoeberlein A. (2019). Exosomes derived from umbilical cord mesenchymal stem cells reduce microglia-mediated neuroinflammation in perinatal brain injury. *Stem Cell Research & Therapy*.

[38] Yang Y., Ye Y., Su X., He J., Bai W., He X. (2017). MSCs-derived exosomes and neuroinflammation, neurogenesis and therapy of traumatic brain injury. *Frontiers in Cellular Neuroscience*.

[39] Sukumaran V., Tsuchimochi H., Fujii Y. (2018). Ghrelin pre-treatment attenuates local oxidative stress and end organ damage during cardiopulmonary bypass in anesthetized rats. *Frontiers in Physiology*.

[40] Li D., Zhang D., Tang B. (2019). Exosomes from human umbilical cord mesenchymal stem cells reduce damage from oxidative stress and the epithelial-mesenchymal transition in renal epithelial cells exposed to oxalate and calcium oxalate monohydrate. *Stem Cells International*.

[41] Zheng J., Min S., Hu B., Liu Q., Wan Y. (2019). Nrdp1 is involved in hippocampus apoptosis in cardiopulmonary bypass-induced cognitive dysfunction via the regulation of erbb3 protein levels. *International Journal of Molecular Medicine*.

[42] Kumar V. (2019). Toll-like receptors in the pathogenesis of neuroinflammation. *Journal of Neuroimmunology*.

[43] Jana M., Palencia C. A., Pahan K. (2008). Fibrillar amyloid-beta peptides activate microglia via tlr2: implications for Alzheimer's disease. *Journal of immunology*.

[44] Reed-Geaghan E. G., Savage J. C., Hise A. G., Landreth G. E. (2009). CD14 and toll-like receptors 2 and 4 are required for fibrillar a{beta}-stimulated microglial activation. *The Journal of Neuroscience*.

